# Experimental But Not Sex Differences of a Mental Rotation Training Program on Adolescents

**DOI:** 10.3389/fpsyg.2016.01050

**Published:** 2016-07-12

**Authors:** Antonio Rodán, María José Contreras, M. Rosa Elosúa, Patricia Gimeno

**Affiliations:** ^1^CEU-San Pablo UniversityMadrid, Spain; ^2^Universidad Nacional de Educación a DistanciaMadrid, Spain; ^3^Spanish Ministry of EducationMadrid, Spain

**Keywords:** mental rotation, mathematics, training, adolescents, sex differences

## Abstract

Given the importance of visuospatial processing in areas related to the STEM (Science, Technology, Engineering, and Mathematics) disciplines, where there is still a considerable gap in the area of sex differences, the interest in the effects of visuospatial skills training continues to grow. Therefore, we have evaluated the visuospatial improvement of adolescents after performing a computerized mental rotation training program, as well as the relationship of this visuospatial ability with other cognitive, emotional factors and those factors based on the experience with videogames. The study, which was performed on students aged 14 and 15 years old, showed a significant improvement in this visuospatial skill for a training group (*n* = 21) compared to a control group (*n* = 24). Furthermore, no significant sex differences were obtained for spatial ability or for any of the other tasks evaluated, either before or after training. Regarding the relationship between skills, a significant correlation between experience with video games and spatial ability was found, as well as between mathematical reasoning and intelligence and with spatial ability in the initial phase for the total sample. These findings are discussed from a cognitive point of view and within the current sociocultural context, where the equal use of new technologies could help reduce the visuospatial gap between sexes.

## Introduction

Visuospatial processing is a key tool necessary to achieve a good performance in certain everyday activities, academic work and professional areas (Halpern et al., 2007), and predicts the success in the STEM disciplines – *Science, Technology, Engineering, and Mathematics* – (Wai et al., 2009). Therefore, the scientific community has shown in recent decades a strong interest in the study of the improvement of visuospatial abilities. Several meta-analyses have reported that repeated practice and training with visuospatial material can improve performance of this skill (Baenninger and Newcombe, 1989, 1995; Marulis et al., 2007; Uttal et al., 2013).

Most research on training of visuospatial skills has focused on adults (e.g., university students), being this type of training on early childhood and adolescence less frequent (see Uttal et al., 2013 for a data review). Adolescence is a key stage of development and learning that can be decisive in a person’s options for a future academic career. In addition, in this stage, the development of spatial vision helps the acquisition of knowledge and the interaction with the physical world (Boletín Oficial de la Comunidad de Madrid [BOCM], 2007, p. 118). For this reason, any intervention with these cognitive skills at this stage or before may be relevant for the academic and professional development of the individual.

Mental rotation (MR) is probably one of the visuospatial skills that has received most attention, as it can be approached from both, psychometric and cognitive perspectives (Sanz de Acedo Lizarraga and García Ganuza, 2003). Furthermore, MR ability and its relationship with academic performance are of particular interest in the STEM disciplines, which often involve tasks such as technical drawing, solving geometric problems, or representing molecular structures, that require a strong visuospatial sense to understand the dynamics and spatial transformation of the structures and objects (Maeda and Yoon, 2013). According to the classification of visuospatial skills developed by Linn and Petersen (1985), MR is conceived as the ability to rotate 2D or 3D figures quickly and accurately in the imagination.

Regarding the training of this spatial ability, several studies have evaluated the degree of improvement amongst the adult population through the use of tasks associated with MR (Larson et al., 1999; Wiedenbauer et al., 2007; Wright et al., 2008; Adams et al., 2014; Meneghetti et al., 2016). Training with tasks related to object rotation has also been studied in puberty and adolescence, although to a lesser extent than amongst the adult population. Overall, these interventions have proved useful, directly improving MR performance (Sanz de Acedo Lizarraga and García Ganuza, 2003; Neubauer et al., 2010; Bergner and Neubauer, 2011) or other related skills, such as visualization (Vz, from now on; Kwon et al., 2002; Rafi et al., 2008; Samsudin et al., 2011). In this type of studies on a young population, the inclusion of a control group – i.e., that has not received any type of training – has not always been considered (Kwon et al., 2002; Rafi et al., 2008; Neubauer et al., 2010; Bergner and Neubauer, 2011; Samsudin et al., 2011), an aspect which influences the knowledge of the real effectiveness of these interventions. To date, only one study by Sanz de Acedo Lizarraga and García Ganuza (2003) on 14 year-old students has evaluated how training using a printed MR task with manipulative support enhances this ability and is transferred to other types of Vz tasks. This study did consider the inclusion of a control group which did not undergo any intervention.

Much of the literature on sex differences in the field of sciences has focused on differences in the rates of female and male students who choose educational paths leading to STEM (e.g., Halpern et al., 2007) disciplines. The relationship of performance in spatial tasks with different areas of science, and the sex differences in the STEM domains has led to the study in recent decades of the average difference in performance between sexes in tasks with a visuospatial content. In comparison with other sub-factors of spatial ability, MR yields greater sex differences, although its magnitude seems to vary according to the different studies (Maccoby and Jacklin, 1974; Linn and Petersen, 1985; Voyer et al., 1995; Maeda and Yoon, 2013). In fact, sex differences in MR tasks have been questioned after certain studies on adult population (Larson et al., 1999; Parsons et al., 2004) and adolescents (Pleet, 1991; Sanz de Acedo Lizarraga and García Ganuza, 2003; Neubauer et al., 2010; Bergner and Neubauer, 2011) have shown that such differences are negligible or non-existent. Evidence according to each age group seems variable, as other studies have obtained differences between sexes in spatial tasks at the age of 10 years or at even earlier ages (Hahn et al., 2009; Titze et al., 2010; Frick et al., 2014). Regarding the adolescent population, Pleet’s (1991) doctoral thesis found no sex differences amongst 13 and 14 year-old students through a MR task – Card Rotations Test – before an intervention for the acquisition of transformation geometry concepts. Similarly, Sanz de Acedo Lizarraga and García Ganuza (2003) didn’t find any sex differences either in the MR performance using a task of cubes in different perspectives, or in Vz tasks evaluated through the Differential Aptitude Test-Spatial Relations (DAT-SR) in 14 year-old high school students. Rafi et al. (2008) and Samsudin et al. (2011) evaluated Vz in 15 and 16 year-old students through a Vz task (Middle Grade Mathematical Project, 1983) which consisted of small unit cubes seen from a certain perspective where participants were required to determine which one of the five other objects was the same as the one depicted, but from another view. The scores obtained in this Vz task were very similar for both sexes, finding no sex differences. Moreover, Samsudin et al. used in their study an internet version of the Mental Rotation Test [developed by Chay (2000)], and didn’t find any significant sex differences in this spatial ability. Other studies on 14 and 15 year-old adolescents have shown that male advantage disappears completely when the rotation task is presented in a real three-dimensional model – similar to virtually reality – format (Neubauer et al., 2010; Bergner and Neubauer, 2011).

In relation to the sex differences found in MR visuospatial training, variable results have also been obtained. While Marulis et al.’s (2007) meta-analysis indicated that the improvement in the performance of this ability is equivalent for both sexes, other studies performed on different age groups have suggested that the mean performance after a training program can be more pronounced in females, which leads to the disappearance of sex differences for such ability (e.g., Pleet, 1991; Boulter, 1992; De Lisi and Wolford, 2002; Sanz de Acedo Lizarraga and García Ganuza, 2003; Ehrlich et al., 2006; Wiedenbauer and Jansen-Osmann, 2008; Neubauer et al., 2010; Cherney et al., 2014).

Since researchers have identified sex differences in spatial skills and have seen that these can be critical for both sexes to define their academic and professional careers, studies have been conducted to assess how different factors – as we shall see below – may affect these sex differences in spatial abilities. Contreras et al. (2012) have noted that, frequently, the “sex” variable is considered a causal factor when “sex” is a broad category that entails so many confounding factors that it is nearly impossible to consider it as a causal factor. The studies that have focused on exploring possible potential correlates that might explain individual differences in spatial abilities have included, for example, emotional factors (i.e., Ramirez et al., 2012), strategic, motivational, experiential, and culture based factors, including stereotype activation (i.e., Moè and Pazzaglia, 2006). For example, Ramirez et al. (2012) studied the so-called “spatial anxiety” (“feelings of nervousness associated with spatial activities”) of children aged between 5 and 8 years of age in order to assess whether there may be a critical factor contributing to sex differences in spatial ability. These authors have reported that spatial anxiety may be present from early childhood, adversely affecting MR tasks. In addition, females seem more likely to show greater anxiety when performing spatial tasks. These sex differences in spatial anxiety have also been found in adults performing navigational tasks, where women reported greater anxiety than men (Lawton, 1994). However, there are no studies on the influence of spatial anxiety on the MR performance throughout the adolescent stage.

Another possible mediator that has been researched in relation to average sex differences in visuospatial processing is the experience with computers and video games. In their research, Terlecki and Newcombe (2005) have found in adults that the experience in the use of computers and video games influences sex differences in mental rotation tasks. Similarly, Quaiser-Pohl et al. (2006) have also reported that boys aged between 10 and 20 years have a higher frequency of interaction with video games than girls of the same age group, especially with “action–simulation” games. Moreover, boys achieved a better performance in the MR task than girls, as a group. However, some studies have already shown that practice with video games can reduce or eliminate these sex differences in spatial skills, including MR (Cherney, 2008; Cherney et al., 2014), and these findings encourage the motivation of females to have a greater interaction with this type of videogames.

Another important cultural factor is the influence of stereotypes. Moè (2012) showed that both women and men are affected by the stereotype and the time limit explanations. In another study, Moè (2016) has shown, for the first time, the influence of school context in reducing sex differences in spatial performance. In his study, Moè showed that exposing girls and adolescents to tasks related to spatial reasoning with instructions that controlled the effect of stereotypes resulted in them achieving the same as the mean level of boys in Mental Rotation tasks. These results are of great importance, as spatial training has also been found to help not only tasks that are obviously spatial. For example, it has been shown that MR training helps in the use of maps, navigating through a city, and in useful, everyday tasks from daily life (Pazzaglia and Moè, 2013). Furthermore, as noted by Newcombe and Frick (2010), “to promote spatial thinking in preschools, and in children’s play, integrating spatial content into formal and informal instruction could not only improve spatial functioning in general but also reduce differences related to gender and socioeconomic status that may impede full participation in a technological society”.

Another aspect that has attracted the interest of some researchers is the relationship between visuospatial skills and mathematics (see Mix and Cheng, 2012 for a review). The reason for this interest is in part due to the fact that both skills – individually and together – are essential tools used in several academic and daily life activities. The relationship between these skills has been observed in research, showing that people with better performances in certain spatial reasoning tasks, often also exhibit better performances in mathematical ability tests (e.g., Baenninger and Newcombe, 1995; Delgado and Prieto, 2004; Rasmussen and Bisanz, 2005; Holmes et al., 2008).

Moreover, some studies have evaluated improvements in mathematical performance for early age and young participants through the practice of games related to visuospatial skills, such as those that require interaction with blocks or pieces or number lines (Wolfgang et al., 2001; Fischer et al., 2011). The repercussion that the practice of visuospatial tasks has on mathematical learning throughout the adolescent stage has scarcely been assessed and certain discrepancies have been observed. While some studies have suggested a positive effect on geometry learning through certain tasks, such as computerized programs with *Tangram* puzzles (Olkun et al., 2005) or manipulative tasks with geoboards, puzzles or MIRA sheets (Boulter, 1992), others have not found such effects using similar tasks involving rotations, reflections, translations and concepts of symmetry (Pleet, 1991; Sundberg, 1994).

Regarding the practice with specific MR tasks to assess the degree of improvement in mathematical learning, a recently published study by Cheng and Mix (2014) aimed at children aged between 6 and 8 years, has evaluated, for the first time, whether a MR training can improve performance of addition and subtraction exercises. According to these authors, the results reflect children’s attempts to solve missing-term problems by mentally rotating missing-term equations into a more conventional format (e.g., 2 + ___ = 7 becomes ___ = 7 - 2). Cheng and Mix noted that such findings are indicative of shared cognitive processing that is sensitive to input, thus raising the possibility that more extensive training would lead to more pervasive changes. However, the repercussion that the practice of MR tasks could have on mathematical learning during periods such as puberty and adolescence has not, to our knowledge, been studied to date, and could prove to be extremely interesting. In fact, within the Spanish education system, some of the contents of secondary school mathematics include numerical reasoning, algebra, geometry and graphs, which contribute to an important spatial load (Boletín Oficial de la Comunidad de Madrid [BOCM], 2007, pp. 121–122). More specifically, in the section on acquisition of basic competences for secondary school mathematics, the importance of the development of spatial vision has on this area of education is clearly mentioned (Boletín Oficial de la Comunidad de Madrid [BOCM], 2007, p. 118).

In light of these previous studies, and despite the extensive research performed so far on visuospatial skills, the performance and the degree of improvement achieved for both sexes could be studied through a computerized program of MR intervention on female and male adolescent students, including the relationship of spatial performance with mathematics, emotional factors and gaming experience in this period of development. It is noteworthy that, throughout the school levels that cover adolescence (specifically, in 4th year of Secondary Education of the Spanish education system), students must decide the area that they wish to study (sciences/technology, humanities, and art), which becomes decisive for their future academic trajectory. Therefore, any intervention occurring before or after this stage becomes essential, especially when the presence of females is scarce in certain degrees where mathematics and science – and hence, visuospatial reasoning – are important. To our knowledge, the present study would be the first to be performed on this age group which includes training and assessment of its effects on a wide group of variables that may have a relationship with this ability throughout adolescence. The hypotheses of the study were: (i) based on previous studies showing that MR can improve after training, it is expected that a computerized training program will show increases in this visuospatial ability in a group of adolescents; (ii) according to previous studies, spatial ability performance of the pretest phase may be similar for both sexes or it may be higher for males, as a group; therefore, boys and girls will benefit in a similar way from the computerized training; (iii) on the one hand, a greater gaming experience is expected for males as a group and on the other hand, participants with a greater experience with video games will perform the spatial ability task better; (iv) given the link between visuospatial skills, fluid intelligence and mathematical reasoning, a positive correlation between these three variables is expected; and (v) a negative correlation between spatial anxiety and spatial ability is expected, in the sense that students with a greater anxiety to perform spatial tasks will present a worse performance of the spatial ability task.

## Materials and Methods

### Participants

The study involved 58 students of 3rd year of Secondary Education from the CEU-San Pablo College in Montepríncipe (Madrid). All of their results were not included in the study, as some participants had not completed all of the phases in the experiment. Finally, the results of 45 students aged 14 and 15 years (boys: *n* = 23, *M =* 14.3, *SD* = 0.42; girls: *n* = 22, *M =* 14.3, *SD* = 0.40) with a medium-high sociocultural level were analyzed. The participants were randomly distributed into two groups: Control group, CG (*n* = 24; 12 boys and 12 girls) and Experimental group, EG (*n* = 21; 11 boys and 10 girls). The Ethics committee of the University (UNED) approved the study with written informed consent from all participants. Parents were contacted through their children’s schools, written consent was obtained from them, in accordance with the Declaration of Helsinki.

### Materials

#### Spatial Ability “E” Subtest, EFAI-3 (Factorial Evaluation of Intellectual Abilities; Santamaría et al., 2005)

This is a typified test (*N* = 23793) which has been validated on different age groups from 7 years of age to adulthood. This test reflects an approach to the visual processing factor, “Gv”, and represents the ability to mentally process visual stimuli (rotate, bend, develop, etc.). The test consists of two practice elements and 27 evaluation elements. The test requires the subject to imagine mental movements and transformations, so that a single stimulus (out of four possible stimuli, located on the right) fits into the blank space (single, double or triple) of a reference stimulus located on the left side and that corresponds to a puzzle of two or more pieces. After 6 min of application of the test, participants must stop performing the task even if they have not finished completing the 27 elements. A single point is awarded for each correct answer provided, so that the total score corresponds to the sum of points awarded for the 27 elements. The studies carried out by its authors indicate that the spatial ability of the EFAI-3 presents reliability coefficients of 0.71 (Santamaría et al., 2005).

#### Numerical Ability “N” Subtest, EFAI-3 (Factorial Evaluation of Intellectual Abilities; Santamaría et al., 2005)

This test contains an approach to the quantitative reasoning factor, “*Gq*”, an aptitude related to the ability to manipulate basic mathematical concepts, arithmetic reasoning, problems of daily life and interpretation of tables and graphs with numerical content. The test consists of three practice elements and 24 evaluation elements, where the subject performs a series of mathematical tasks related to arithmetic operations, verbal problems and problems in chart or table format. Only one response (out of four possible) is correct. After completing the established 14 min, participants must stop the execution of the test even if they have not completed the 24 elements. A single point is awarded for each correct answer given, so that the total possible score is 24 points. Studies conducted by the authors of EFAI-3 indicate that this ability has reliability coefficients of 0.77 (Santamaría et al., 2005).

#### Raven’s Standard Progressive Matrices, SPM (Raven et al., 1996)

This test evaluates the ability to deduct relationships, one of the main components of general intelligence. The test consists of 60 sheets spread over 5 series with multiple choice of 6 or 8 response options, so that only one correct option is the best suited to an element located on the top part of the sheet. The elements of each series increase in difficulty as the test progresses, and to which there is no time limit, although its estimated duration is about 60 min. A single point is awarded for each correct answer provided, so the total possible score is 60 points. Its reliability varies depending on the methodology used: two halves (0.90), test–retest (between 0.83 and 0.90), according to Seisdedos (1995).

#### Child Spatial Anxiety Questionnaire (CSAQ Adapted from Ramirez et al., 2012)

This is an adapted version of CSAQ, developed to assess possible spatial anxiety in children aged between 5 and 8 years. The questionnaire contains eight questions about some spatial activities that may make them nervous in their everyday life (“How do you feel when a friend asks you how to get from school to your house?”), in their academic life (e.g., “How do you feel when your teacher asks you whether these shapes are rectangles and why?”) or in other specific activities in which they have to perform spatial tasks in a certain amount of time (e.g., “How would you feel if your teacher asked you to build this house out of these blocks in 5 min?”). In our study, the original questionnaire was adapted according to several considerations: (a) the Spanish language; (b) age, so as to better suit the age group of our study; (c) culture, to adequately fit the context of our country, and (d) scoring criteria, using a 5-level *Likert*-type scale for which the score ranged from 0 to 2 points (0 points, equivalent to “I don’t get nervous”; 0.5 points for “I get a little nervous”; and so on, up to level 5, which is equivalent to 2 points “I get very, very nervous”). Moreover, to clarify the definition of “nervousness” and to ensure an adequate use of the *Likert* scale, it was specified that nervousness referred to the level of frustration or difficulty that a specific task of those contained in the questionnaire could elicit. In order to obtain the final score of the CSAQ-adapted (CSAQ-a), the total number of points obtained was considered; so that the minimum anxiety score is 0 points and the maximum is 16 points. The authors of this questionnaire have found a reliability of 0.56 (Cronbach’s alpha) in their study with participants between 5 and 8 years of age.

#### Videogame Experience and Preference Questionnaire

It is a questionnaire adapted from some of the questions used in Section B of the Survey of Spatial Representation and Activities (SSRA; Terlecki and Newcombe, 2005) and in the self-report questionnaire on Computer-game experience (Quaiser-Pohl et al., 2006). The questionnaire presented five questions that assess the participant’s type of commitment to video games. The questionnaire questions relate to: (i) whether or not they play; (ii) whether they like playing; (iii) the frequency with which they play; (iv) if they believe they are skilled at it or not, and (v) which is their favorite game. With the exception of question 4 (of a subjective nature) and question 5 (which requires a qualitative analysis), questions 1–3 are taken into consideration when calculating the final score. In Question 1, no points are awarded if the participant does not play video games, and 1 point if, on the contrary, he states that he does play video games; in Question 2, no points are awarded when the participant does not like video games and 1 point if he states that he likes them; in Question 3, for the answers of “one day a week”, “at weekends”, “3 days a week” and “every day of the week”, 1, 2, 3 and 4 points are awarded, respectively. Therefore, the minimum dedication score is 0 points and the maximum is 6.

#### Mental Rotation Training Program (MRTP)

**FIGURE 1:**
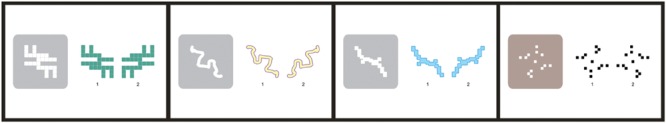
**Example items from the Mental Rotation Training Program (MRTP).** In all examples, the first stimulus is rotated 180° and the second stimulus is flipped over the *X* axis with respect to the target.

In order to adequately adapt the MR task to a design suitable to our purposes – age group of interest and calibration of some of the factors of the task – a pilot study was performed. The stimuli were designed specifically for the study using the Corel Draw Graphics Suite X3 computer software. Each slide consisted of: (i) a *target* that simulates an empty mold located on the left side of the slide and inside a gray box, and (ii) another two figures located on the right side, listed as “1” and “2”, which have the same shape as the target, but with a specific filling and are orientated differently from each other and the *target.* The figures have a flat perspective with geometric or linear shapes (**Figure 1**). The task requires the subject to imagine rotations to decide whether either of the two stimuli “1” and “2” on the right fit into the *target* on the left when they are turned or rotated (not flipped or mirrored). In each slide, either only one of the figures, both figures or none of the figures will fit into the target mold after being turned or rotated (these possibilities were counterbalanced in each session). The orientation of the rotated figures could be 90°, 180° or 270°, and the figures that do not fit the target were flipped across the *X* axis, the *Y* axis, the *X* axis plus an additional 90° rotation or the axis *X* plus an additional 270° rotation. All these were counterbalanced across sessions. Furthermore, the difficulty within each session and between sessions was increased by manipulating the degrees of rotation and the complexity of the stimuli.

### Procedure

**FIGURE 2:**
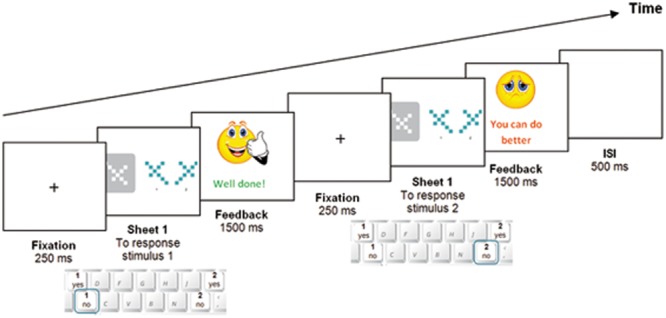
**Example of the temporal sequence of a MRTP trial**.

The E-Prime computer software version 1.0 (Psychology Software Tools, 2002) was used for the programming, presentation of stimuli and data collection of the MRTP. Only the EG participants carried out the MRTP in three different sessions spread over three consecutive days – 1 session per day –. During the task, the participants had to decide whether stimulus 1 fits into the standard target, answering “1 YES” or “1 NO”. Afterwards, participants had to do the same with stimulus 2, answering “2 YES” or “2 NO”. Participants recorded their responses through the computer keyboard. Each session consisted of two phases: (i) a practice round with 10 slides, in which each response was followed by feedback on whether it was correct (happy face and “well done”) or incorrect (sad face and “you can do better”), and an additional animation with the progressive rotation of each figure, confirming whether or not they fitted into the target, to strengthen the understanding of the task; and (ii) a specific training round of 100 slides per session in which participants did not visualize the animated verification sequence, but still received feedback on whether their answer was right or wrong (**Figure 2**). The approximate length of each session was around 40 min so that at the end of the third session, participants had completed a total of 330 slides in approximately 120 min of training, varying according to each participant, as there was no limit in the response time. The MRTP was carried out collectively in the school’s computer room, and each student performed the task individually. The monitor model was the same for all participants and the screen resolution was set to 1280 × 1024 so as to maintain a regular format (unstrained) and to comply with the resolution required by the E-Prime program. The approximate working distance was about 50 cm. and the environment in the computer room remained quiet and free from noise and distractions.

#### Pretest

The first day was performed in the exam room with three examiners, and the tables were arranged individually to avoid any kind of plagiarism in the responses of the participants. Participants were observed discreetly to check that the answers were collected in the correct form. The order of the tasks for both groups was as follows: Raven’s Progressive Matrices, SPM version (Raven et al., 1996), spatial “E” and numerical “N” subtests of the EFAI-3 (Santamaría et al., 2005), Child Spatial Anxiety Questionnaire (CSAQ adapted from Ramirez et al., 2012) and Videogame Experience and Preference Questionnaire.

#### Training

The EG carried out the MRTP in the computer room throughout days 2, 3, and 4. The approximate duration of training was about 40 min per session, totaling approximately 120 min of training. The CG remained in their break time (recess) while the training program was conducted.

#### Posttest

A final session was carried out, under the same conditions and the same tests as the pretest phase, except for the Videogame Experience and Preference Questionnaire (VEPQ), which was not necessary to repeat.

## Results

The descriptive statistics of the different variables evaluated in both groups are shown in **Table 1**

**Table 1:** Mean scores and standard deviations of the two groups (control and experimental group) for the different tests used in both phases of the study.

Tests	Control group (*N* = 24)				Experimental group (*N* = 21)			
	Pretest		Posttest		Pretest		Posttest	
	*M*	*SD*	*M*	*SD*	*M*	*SD*	*M*	*SD*
EFAI-E (max. = 27)	9.21	2.13	12.63	3.87	9.86	3.10	15.33	3.87
EFAI-N (max. = 24)	12.88	4.11	14.46	3.61	15.95	3.65	16.24	3.08
Raven (max. = 60)	49.54	6.21	49.42	5.14	49.29	4.51	50.62	4.62
CSAQ-a (max. = 16)	4.17	1.53	3.92	1.95	4.48	2.00	3.88	1.81
VEPQ (max. = 6)	3.63	1.50	–	–	4.10	1.22	–	–

*EFAI-E – Evaluación Factorial de las Aptitudes Intelectuales–Espacial (Factorial Evaluation of intellectual abilities–Spatial ability “E”); EFAI-N – Evaluación Factorial de las Aptitudes Intelectuales–Numérico (Factorial Evaluation of intellectual abilities–Numerical ability “N”); CSAQ-a – Child Spatial Anxiety Questionnaire (adapted from Ramirez et al., 2012); VEPQ – Videogame experience and preference questionnaire; VEPQ was evaluated only in the pretest.*

Four MANOVAs 2 (group: experimental vs. control) × 2 (sex: males vs. females) × 2 (time: pretest vs. posttest) were run, respectively on EFAI-E, EFAI-N, Raven, CSAQ-a, with the first two factors between-subjects and the last within-subjects, and using the *Greenhouse-Geisser* correction (the most conservative). In order to contrast hypotheses 1 and 2, the spatial ability results (EFAI-E) revealed a significant main effect due to time factor, *F*(1, 41) = 90.09, RMS = 441.98, *p* < 0.001, *η*^2^= 0.69 and an interaction between time × group, *F* (1, 41) = 4.82, RMS = 23.65, *p* < 0.05, *η*^2^ = 0.11. The Tukey critical value was 0.943. No other significant effects emerged on EFAI-N, Raven, CSAQ-a.

A statistically significant improvement was observed for the EG after the intervention in spatial ability (EFAI-E), *t* (20) = 8.95, *p* < 0.001, Cohen *d* = 1.56. The CG showed a significant increase in the posttest phase with respect to the pretest phase in spatial ability (EFAI-E), *t* (23) = 5.12, *p* < 0.001, *d* = 1.09. The increases in spatial ability were significantly higher in the EG compared to the CG, *t* (43) = 2.25, *p* < 0.05, *d* = 0.68, [EG: *M* = 5.48, *SD* = 2.80; CG: *M* = 3.42, *SD* = 3.27], confirming the main hypothesis of the study.

In the EG, no significant sex differences were found for the increases in spatial ability (EFAI-E), *t* (19) = 0.12, *p* = 0.91 [boys: *M* = 5.55, *SD* = 3.11; girls: *M* = 5.40, *SD* = 2.59].

Furthermore, in order to contrast hypothesis 3, an ANCOVA 2 (groups) × 2 (sex) was performed for the EFAI-E scores of the pretest phase, considering previous experience with videogames as a covariate. No significant differences were found between groups, or between sexes and no significant differences were found for experience with videogames. The same ANCOVA was performed for the EFAI-E scores after training, not finding any significant differences.

For the total sample, no significant differences were obtained between sexes for the experience with video games (VEPQ), *t* (43) = 0.12, *p* = 0.90 [boys: *M* = 3.87, *SD* = 1.42; girls: *M* = 3.82, *SD* = 1.37].

Regarding the contrast of hypotheses 4 and 5, in the pretest phase for the total sample, a significant positive correlation between experience with video games and spatial ability (*r* = 0.30, *p* < 0.05) was found, in the sense that participants with a greater experience with video games obtained a better performance in the spatial ability task. Moreover, significant positive correlations between numerical ability and general intelligence (*r* = 0.37, *p* < 0.01) and with spatial ability (*r* = 0.26, *p* < 0.05) were also obtained. The correlations between the other variables for the total sample in the pretest phase and the post-test phase in EG were not significant, hence not confirming in this study the prediction about the expected relationship between spatial ability and anxiety.

## Discussion

The main aim of our study was to evaluate whether an inter- vention program in mental rotation (MR) improved this skill in a group of adolescent students. To do this, we designed a mental rotation training program and we applied it to an experimental group (EG), comparing the degree of improvement in a different spatial ability task in relation to a control group (CG). The results indicated that the training of MR produced an improvement of this visuospatial ability. The EG showed the largest increases in test scores in the posttest phase in relation to the CG and the difference in increase between the two groups was statistically significant.

It is worth mentioning some important aspects of this study: one is the fact of including a control group (which did not undergo any intervention), which is an issue that has not always been included in other studies (Kwon et al., 2002; Rafi et al., 2008; Neubauer et al., 2010; Bergner and Neubauer, 2011; Samsudin et al., 2011), and is important to better understand the effectiveness of these interventions. Another aspect is that the task used during training was different from the task used in the evaluation, an aspect which has not always been considered in previous studies (see Uttal et al., 2013 for a review). Certain studies performed on teenagers have carried out intervention tasks related to rotation, translation, reflection, symmetry and visualization of objects, but they have not used strictly MR tasks, a detail that seemed interesting for us to include in the evaluation of participants of this age group. Sanz de Acedo Lizarraga and García Ganuza’s (2003) study did consider these aspects, performing an intervention to improve MR through printed tasks and cube manipulation. In our study, we decided to include a computerized intervention program that would be similar to a video game, with the aim of further motivating participants in this age group. In fact, the positive impact that video games can have on the improvement of MR was taken into consideration (Okagaki and Frensch, 1994; De Lisi and Cammarano, 1996). Moreover, the feedback technique (in which a message is received after each response to indicate the correctness of the response) is an efficient methodological strategy that affects future performance of mental rotation (e.g., Kyllonen et al., 1984; Kass et al., 1998) by decreasing the number of errors and the reaction time. In this sense, the design of our training program could have had a motivational effect on the experimental group.

The absence of significant sex differences before training contrasts with previous studies that highlight an advantage for males as a group, especially in MR tasks (Maccoby and Jacklin, 1974; Linn and Petersen, 1985; Voyer et al., 1995). Nevertheless, our results are in line with studies that have shown that the mean differences between sex groups in this type of cognitive tasks are becoming smaller (Pleet, 1991; Larson et al., 1999; Sanz de Acedo Lizarraga and García Ganuza, 2003; Parsons et al., 2004; Hyde, 2005; Neubauer et al., 2010; Bergner and Neubauer, 2011). We would like to discuss some aspects that explain our results with respect to this hypothesis of average differences between sexes. One aspect is that MR tasks have yielded a greater effect size in terms of sex differences, compared to other visuospatial factors such as spatial relationships or Vz (Voyer et al., 1995). In addition, the effect size in MR seems to vary depending on the type of task used (Voyer et al., 1995); for example, tasks in three-dimensional format, such as the MRT (Shepard and Metzler, 1971; Vandenberg and Kuse, 1978; Peters et al., 1995), which presents a substantially higher effect size than that of other tests like the *Primary Mental Abilities Test* (PMA; Thurstone, 1938) or the *Cards Rotation Test* (Ekstrom et al., 1976). As for the assessment test that we included in our study to evaluate spatial ability (Spatial ability “E” subtest, EFAI-3; Santamaría et al., 2005), the authors have considered it as a test that evaluates the ability to mentally imagine transformations and rotations of an object, the retention of information in working memory and its integration with other additional information. In this sense, our task could be adjusted to the Vz factor, which has been defined as the ability to mentally rotate, twist, or invert a pictorially presented stimulus object where the underlying ability seems to involve a process of recognition, retention, and recall of a configuration of an object manipulated (McGee, 1979). Therefore, the two-dimensional design of the EFAI “E” – similar to PMA or CRT – and its potential demand of Vz could explain, at least in part, these results. The reason we chose this test is because it allows to evaluate spatial ability in children and all through to adulthood, and this research has been carried out in parallel with another study performed on children in primary education, in order to assess individual differences related to age (Gimeno et al., under review). While Vandenberg and Kuse’s (1978) MRT or Peters et al.’s (1995) version have been used on adolescents, these tests are not frequently used in a 3D format on young children (e.g., elementary-school children), yielding poor performance for a MRT with 2D figures (Neuburger et al., 2011; Hoyek et al., 2012) similar to those in PMA, CRT or EFAI-E. In fact, Hoyek et al. (2012) have noted the difficulty of applying the MRT V-K on 7- and 8-year-old children. Our EFAI-E task is a typified test (*N* = 23793) which has been validated on different age groups from 7 years of age to adulthood. Furthermore, although the EFAI-E spatial task requires the participant to put into play a mental rotation, the task differs from that used in our training, which allows an assessment of the effects of training on tasks not identical to those trained. Regarding the timing of the spatial task, we would like to point out that some studies with MR tasks (original MRT by Vandenberg and Kuse, 1978; redrawn MRT version by Peters et al., 1995) have shown that sex differences are minimized and even disappear when the conditions of the task are performed with any time constraint (e.g., Goldstein et al., 1990; Glück and Fabrizii, 2010). However, these results don’t seem to be conclusive, as some studies that have manipulated time conditions – e.g., short period and unlimited time on the MRT – have shown no evidence that the sex difference on the MRT was affected by the time conditions (Masters, 1998). Similarly, in Peters (2005) study, it was observed that the scores in the MRT task increased for both men and women, but there was no significant reduction in the magnitude of sex differences after extending the time limit to perform the MRT. It is interesting to note that in our study, in spite of using a spatial task under limited time constraints during the pre and post-test phases, no sex differences have been found.

The literature has considered numerous factors (biological, social, and cognitive, based on experience and practice, or based on the intrinsic characteristics of the stimuli used in the MR task) to explain the average differences between sexes. Maccoby and Jacklin (1974) found that some sex differences in spatial ability occurred in favor of girls at an early age, with a reversal of the effect as they come into adolescence. Similarly, Bergner and Neubauer (2011), based on the results obtained by Geiser et al. (2008), offered a possible explanation for the increase of sex differences based on greater participation of men, as a group, in more spatial activities of everyday life that women – experience factor –, which could suggest that environmental factors or experience may influence the development of spatial abilities. However, we believe that the interaction of the female gender with new technologies, such as tablets, smartphones or video games, may be contributing to minimize average sex differences in spatial processing. In fact, research has already shown that there are no sex effects when the MR tasks are presented within a technological environment (Larson et al., 1999; Parsons et al., 2004; Neubauer et al., 2010). Similarly, our results follow this line regarding experience with video games, where we found no differences between male and female adolescents in the use of these technologies, which may have contributed partly to the lack of sex differences in spatial ability found in this study.

In relation to this, one of our initial hypotheses was that male students would have a greater gaming experience that female students and those students with a greater experience in video games would have a better performance in the spatial ability task. Indeed, male students showed a slightly higher preference and experience in video games, but there was no statistically significant difference between sexes. As we have mentioned above, this non-significant difference could indicate that females, as a group, increasingly interact more with video games. Our results are in line with those obtained by Terlecki et al. (2011), who found almost as many similarities as differences between sexes in their gaming preference. Their results showed that both, males and females, use similar consoles, enjoy adventures games and want funny games. Terlecki et al. (2011) concluded that the gap between sexes, favoring males, may be decreasing with current technologies, and the present study confirm this tendency. In fact, although we did not analyze the interaction with the type of video game, a preliminary analysis of the questionnaire’s responses showed that female students exhibited a clear trend towards logic games and puzzles (e.g., Candy Crush), which may require certain cognitive and spatial skills. This finding could be in line with the results obtained by Quaiser-Pohl et al. (2006), who observed that women had a greater preference for logic games and skill training. Regarding the effect of playing videogames on spatial performance, no such effects were found across groups or across sexes. However, the correlation analysis performed for the total sample confirmed that those participants with a greater interaction with video games were those with a significantly better performance in the spatial ability task. These results are similar to those obtained in previous studies showing how some games like “Tetris” or “Blockout” require spatial processes such as MR and Vz (Okagaki and Frensch, 1994; De Lisi and Cammarano, 1996).

Another aspect analyzed in this work was the possible relationship between spatial ability, intelligence and mathe matics. In our study, the results did show a correlation between spatial ability and mathematics in the pretest phase for the overall sample. These results are in line with previous research that have noted that individuals with better performances in certain spatial reasoning tasks, often have a better performance in mathematical ability tasks (e.g., Baenninger and Newcombe, 1995; Delgado and Prieto, 2004; Rasmussen and Bisanz, 2005; Holmes et al., 2008). Other authors (Thompson et al., 2013) have found a significant correlation between MR and basic numerical skills – numerical comparison task and number line mapping task – in adults. However, it is worth noting that numerical ability is only one component of mathematical ability, and the mathematic test used in our study – with adolescents – may involve more complex functions that go beyond this type of numerical representations. In fact, we have evaluated the mathematical ability through three types of tasks: numerical operations, verbal problems (with statements) and problems with numerical information such as graphs or tables. It is possible that each type of math exercise does not require the visuospatial ability in the same way. As noted by Mix and Cheng (2012), certain spatial tasks could be more related to specific mathematical tasks due to the similarity of the processes.

Regarding the link between spatial ability and intelligence, we have not found any relationship, contrary to what has been obtained through factorial studies, which have shown that spatial tests are good measures of the *g* factor (e.g., Lohman, 1996). It is possible that this disparity may be due to the task used in our study to evaluate spatial ability. On the one hand, it is common for studies to include MR tasks, such as *PMA, MRT*, or *CRT*, which could be different in the psychometric context of the EFAI-E spatial task, although they all demand MR. On the other hand, the studies carried out by the authors of EFAI-3 (Santamaría et al., 2005) show that this spatial subtest presents moderate correlations (0.41) with the Raven SPM parallel test. Santamaría et al. (2005) indicated that “the magnitude of these correlations can be influenced by the application conditions of both tests, in the case of EFAI under time limit conditions and in Raven SPM under power conditions (no time constraints, each subject can use whatever time they require). If the correlation between the Raven and the EFAI scores is studied under power conditions (...), this correlation increases” (Santamaría et al., 2005, p. 80). In any case, the correlation between these skills obtained in our study is far from that provided by these authors. Therefore, its evaluation would be interesting to include in future studies to better understand the nature of this correlation.

As for the results of the correlation between mathematical ability and intelligence, they showed a significant correlation (*r* = 0.37), which is in line with the recent study by Pina et al. (2014) on participants aged between 9 and 13 years.

Another variable of interest in the study was the “spatial anxiety”, which has been recognized as a factor influencing sex differences in spatial ability (Ramirez et al., 2012). After the intervention, the EG showed a significant decrease in spatial anxiety, something that did not happen for the CG. This effect is not surprising, as the spatial experience that the MR training provides could have translated into greater self-confidence to perform certain spatial tasks within the CSAQ questionnaire. The group of female students in the total sample and in the pretest showed a slightly higher spatial anxiety than male students and after the intervention, the EG students decreased their anxiety even further, but no significant sex differences were found in any case. In relation to the link between the spatial ability and spatial anxiety, our results showed a negative correlation between these two variables, in the sense that students with a higher spatial anxiety showed a worse performance in spatial ability. However, contrary to that hypothesized, this correlation was not significant. These results differ from those obtained by other authors in studies with young children (Ramirez et al., 2012) and adults (Lawton, 1994), which show a correlation between anxiety and spatial ability, and sex differences in anxiety. These discrepancies with respect to previous studies may be due to the nature of the task used to assess spatial anxiety. For example, Lawton’s study based on spatial anxiety to navigate in an environment and carry out certain orientation strategies, is very different from the evaluated spatial ability task and from the situations raised in the spatial anxiety questionnaire in our study. Although the spatial anxiety questionnaire we use is an adaptation of a short questionnaire used by Ramirez et al. (2012) on the degree of difficulty a person has in certain situations in a spatial context, the results we have obtained also differ from those reported by these authors. This could be due to the difference in age between both studies (5–8 years for Ramirez et al. and 14–15 years in our study) and to the interpretation given by the children of the “nervousness” – termed this way by these authors – generated by a specific spatial task. In our age group, we preferred to use the word “dificultad (difficulty)” to perform a specific task. Furthermore, it is possible that some of the questions in the questionnaire do not refer to a purely spatial situation, implicating other skills such as mathematics (e.g., indicating the bisector of a segment, angles and distances between points), verbal (e.g., giving an explanation of how to get to a particular place) or general knowledge (e.g., marking a location on a geographical map). In our opinion, the inclusion of questions on more specific spatial situations (e.g., ease to imagine object rotations, to visualize different perspectives, etc.) or situations of everyday life [e.g., ease to understand a map or construction/DIY (Do It Yourself) instructions without having to rotate the information given] could provide additional spatial aspects to this questionnaire.

With regard to the contributions of this study, we would like to note that this work may be one of the first researches on adolescents that analyses the degree of improvement offered by a computerized training with specific MR tasks and the relationship of this spatial ability with mathematics. Furthermore, we included a control group – i.e., a group that does not receive any training –, an aspect which has not always been considered in this age group. In addition, some factors that could be involved in possible sex differences (which were not found) in spatial ability were evaluated. The results presented here demonstrate that this spatial ability could be improved with training. Finally, this research encourages a better understanding of the training effect of MR on specific areas or mathematical content, and the study of other variables involved in the spatial-mathematical relationship. Therefore, we encourage the scientific community to continue researching in this area of interest.

This study has some limitations, which we would like to highlight, in light of future research. For example, our training task was relatively short. This may lead to effects and generalizability being limited. In this sense, we note the potential importance that the assessment of the durability of the effects produced by training or of the transfer to other spatial activities of daily life may have in the field of cognitive psychology.

## Author Contributions

Conceived and designed the experiment: All authors; Performed the experiment: AR; Analyzed the data: AR. Interpretation of the data: All authors. Drafted the paper: AR. Contribution to the redaction: All authors. Provided critical revision: MC and ME. Approved the final version of the paper for submission: All authors.

## Conflict of Interest Statement

The authors declare that the research was conducted in the absence of any commercial or financial relationships that could be construed as a potential conflict of interest.
